# Efforts to address the Sustainable Development Goals in older populations: a scoping review

**DOI:** 10.1186/s12889-023-15308-4

**Published:** 2023-03-08

**Authors:** Vlada Shevelkova, Calum Mattocks, Louise Lafortune

**Affiliations:** 1 Department of Public Health and Primary Care, University of Cambridge, Forvie Site, Cambridge Biomedical Campus, CB2 0SR Cambridge, England; 2 Cambridge Public Health, University of Cambridge, Forvie Site, Cambridge Biomedical Campus, CB2 0SZ Cambridge, England; 3 Cambridge Public Health, University of Cambridge, Forvie Site, Cambridge Biomedical Campus, CB2 0SZ Cambridge, England

**Keywords:** Sustainable Development Goals, Age-friendly communities, Older adults.

## Abstract

**Background:**

The United Nations *Decade of Healthy Ageing* (2021–2030) seeks to create multisectoral changes that align healthy ageing with the United Nations’ Sustainable Development Goals (SDGs). Given that the SDGs have completed their first five years, the objective of this scoping review was to summarise any efforts launched to directly address the SDGs in older adults in community settings prior to the *Decade*. This will contribute to providing a baseline against which to track progress and identify gaps.

**Methods:**

Following Cochrane guidelines for scoping reviews, searches were conducted in three electronic databases, five grey-literature websites, and one search engine between April to May 2021; and limited to entries from 2016 to 2020. Abstracts and full texts were double-screened; references of included papers were searched to identify additional candidate publications; and data were extracted independently by two authors, using an adaptation of existing frameworks. Quality assessment was not conducted.

**Results:**

In total, we identified 617 peer-review papers, of which only two were included in the review. Grey literature searches generated 31 results, from which ten were included. Overall, the literature was sparse and heterogeneous, consisting of five reports, three policy documents, two non-systematic reviews, one city plan, and one policy appraisal. Initiatives targeting older adults were mentioned under 12 different SDGs, with SDG 1 (*No Poverty*), SDG 3 (*Good Health and Wellbeing*), SDG 10 (*Reduced Inequalities*), and SDG 11 (*Sustainable Cities and Communities*) being the most commonly discussed. Also, SDG-based efforts frequently overlapped or aligned to the eight domains of age-friendly environments outlined in the World Health Organisation framework.

**Conclusion:**

The review has documented the extent, range, and nature of available research and provided an initial evidence backdrop for future research and policy development.

**Supplementary Information:**

The online version contains supplementary material available at 10.1186/s12889-023-15308-4.

## Introduction

The world’s population is ageing fast. By 2050, the global population will increase by two billion [1], and persons aged over 65 – who already form the fastest-growing age group – will outnumber young people aged 15 to 24 [2]. Ageing is a multisectoral challenge with economic, social, and health effects that will put unprecedented pressure on health, welfare, and social care systems [3]. Population ageing has far-reaching implications for our planet, not least as a major driver of population growth that further increases demands on natural resources and ecosystems [4]. This has fundamental impacts on sustainable development efforts to eradicate poverty, achieve food security, and build inclusive, resilient communities. Conversely, population ageing also creates opportunities and societal advantages, as older adults contribute to their communities via work, volunteering, and informal care [5, 6]. Older people are a resource to society that will play an increasingly vital role in the future.

Healthy ageing, a concept adopted by the World Health Organisation (WHO) in 2015, is key to empowering the contribution of older people to society and maximising their well-being throughout life [2]. It is defined as “a process of developing and maintaining the functional ability that enables well-being in older age”, where “well-being” is considered holistically to include happiness, satisfaction, and fulfilment, and “functional ability” denotes the health-related attributes that enable people to be and to do what they value [7]. The WHO affirms that ageing is not a purely biological process: it is influenced by external factors like the built environment, societies and communities, policies, services, and systems [7]. The environments older people live and work in must enable healthy ageing by supporting their autonomy and encouraging greater connectivity, security, and identity [7].

Over the last 20 years, there have been many international policy developments related to healthy ageing, including the *Madrid International Plan of Action on Ageing* (2002) [8], *Active ageing: a policy framework* (2002) [9], the *World report on ageing and health* (2015) [7], and the *Global strategy and action plan on ageing and health* (2017) [10]. Concomitantly, international policy has focused on sustainable development, with the United Nations (UN) introducing the *2030 Agenda for Sustainable Development* in 2016. The *Agenda* outlined 17 Sustainable Development Goals (SDGs), that aim to eradicate poverty and hunger, protect the planet, foster peace and justice, and mobilise partnerships for sustainable development [11, 12]. The most recent policy development, the UN *Decade of Healthy Ageing* (2021–2030), seeks to attain both healthy ageing goals and SDGs by empowering strategies proposed by its predecessors (like the development of age-friendly environments and systems for long-term care [7, 10]) and encouraging multisectoral action on healthy ageing [13]. Specifically, the *Decade* aims to transform four areas of action and 11 SDGs related to healthy ageing [13]. Prior to this development, there has been a limited focus within the SDGs on older adults – very few goals mention older adults, with the group often being referenced alongside other vulnerable populations like children and people with disabilities. Therefore, the *Decade* is unique, tying together healthy ageing and SDGs for the first time, to make the Goals actionable and relevant to older adults.

The *Decade* has brought attention to healthy ageing and highlighted its importance to meeting the SDGs. But the SDGs have already completed their first five years. Have any recommendations, policies, interventions, or indicators addressed the SDGs in older populations prior to the *Decade*? This scoping review investigates this question. Specifically, it examines the extent, range, and nature of research that addresses the SDGs in community settings, with a focus on older adults.

The focus on community settings stems from the ongoing importance of the concept of “age-friendly cities and communities” within international policy documents [14]. Environments, specifically communities, are thought of as being central to healthy ageing as they promote health and eliminate barriers – this is demonstrated by the renewed commitments to enhance the *Global Network for Age-friendly Cities and Communities* within the *Decade*. For the purposes of the review, a community was defined as “a directly elected or mandated public governing body possessing within a given territory, as defined by law, a set of competences to deliver public goods and services to citizens; inclusive of sub-national organisational levels from the provincial or state level, to villages and townships with limited population numbers”. This is the definition used in the Terms of Reference for the membership in the *Global Network for Age-friendly Cities and Communities* [15].

## Methods

In keeping with the Cochrane guidelines, we chose a scoping review approach because the literature has not previously been comprehensively reviewed and is heterogeneous in nature, consisting of peer-reviewed literature, international and national policy documents, and policy appraisals [16]. Searches were conducted between April and May 2021; peer-reviewed literature searches (in Scopus, Medline, and Global Health databases) were followed by grey literature searches (in Google and on the websites of the UN, WHO, Centre for Ageing Better, International Federation of Ageing, and Organisation for Economic Co-operation and Development). The searches were limited to a four year period, running from the introduction of the SDGs (in January 2016 [11]) to the start of the *Decade of Healthy Ageing* (in January 2021 [17]). Grey literature searches were limited to the first 100 results. Table 1 depicts the search strategy; the searches were adapted for each database to be reproducible.

**Table 1:** Search strategy for the scoping review

Themes	Search Terms
Theme 1, Population:	(*aged OR retir* OR *age OR ageing OR *aging OR *old OR elder* OR senior* OR pension*) AND
Theme 2, Concept:	(SDG* OR “sustainable development goal*”) AND
Theme 3, Context:	communit* OR city or cities or town* OR village* or neighbourhood* OR residence
Limits:	English Language; published between 2016 to 2020; humans

*Notes*: The timeline was chosen due to the introduction of the Sustainable Development Goals in January 2016 [11] and launch of United Nations *Decade of Healthy Ageing* in 2021 [17]

Abbreviations: SDG = Sustainable Development Goal

First, titles were screened by VS; then, abstracts and full texts were double screened by VS and CM, with discrepancies resolved via discussion. After screening and inclusion of papers, reference lists of the peer-reviewed articles were searched to avoid any data being omitted; also, forward searches were conducted to identify relevant papers that referenced the peer-reviewed articles.

**Fig. 1:**
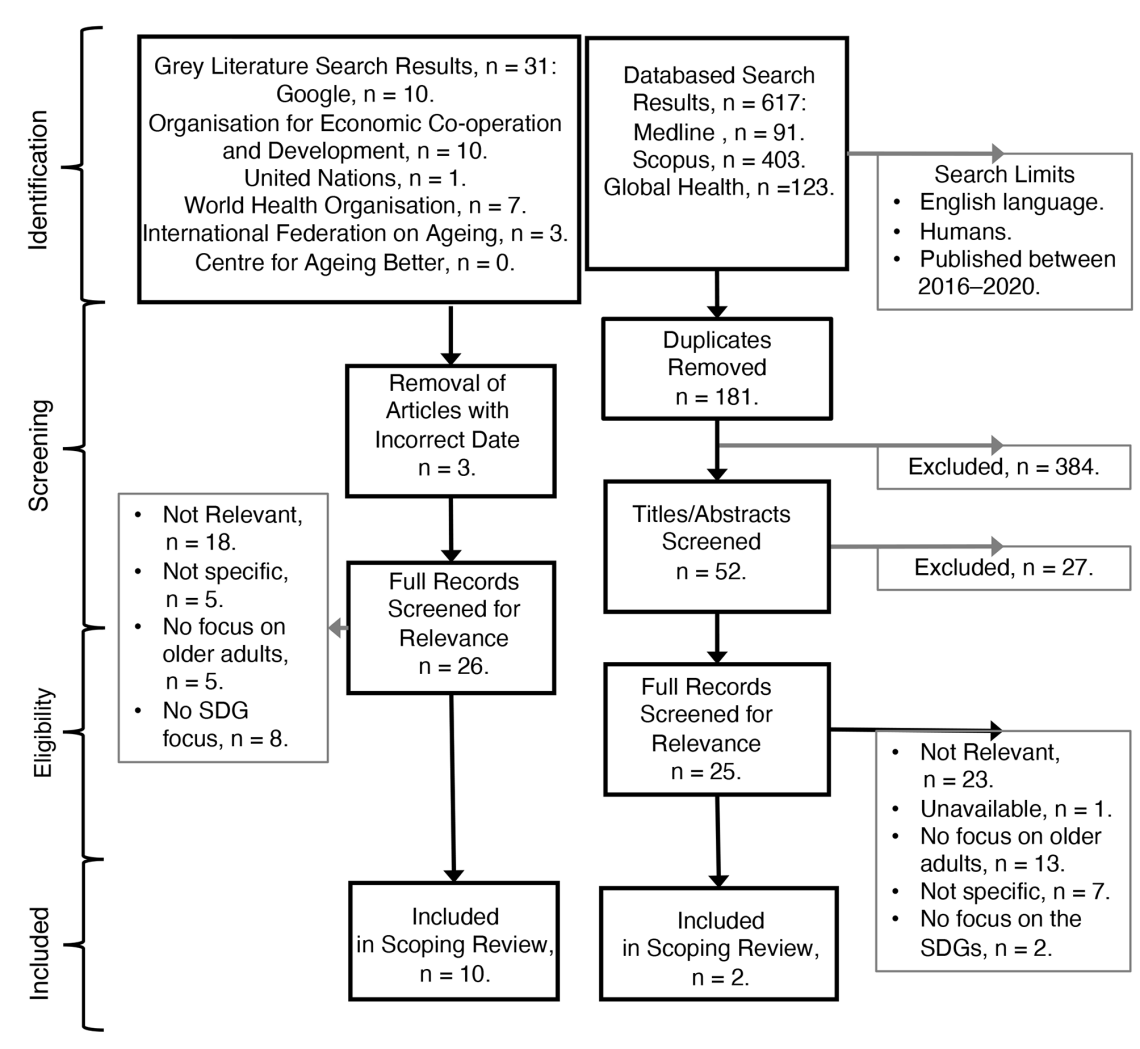
Flow diagram of the study selection strategy

Extraction of data from each document or study was streamlined using adaptations of existing frameworks [18]. We summarised the results narratively and descriptively to align with the objectives of the review. Quality assessment is not a standard procedure for scoping reviews [16] and was not conducted. However, a SPIDER search tool, which determined the inclusion and exclusion criteria for the review, was used (provided in Supplementary Table 1). The document selection process is shown in Fig. 1.

## Results

Table 2 provides the outlines and titles of the SDGs, to make the results more digestible [19].

**Table 2:** Titles and descriptions of the Sustainable Development Goals

SDG Title	SDG Outline
SDG 1 – No Poverty	End poverty in all its forms everywhere.
SDG 2 – Zero Hunger	End hunger, achieve food security and improved nutrition, and promote sustainable agriculture.
SDG 3 – Good Health and Well-being	Ensure healthy lives and promote well-being for all at all ages.
SDG 4 – Quality Education	Ensure inclusive and equitable quality education and promote lifelong learning opportunities for all.
SDG 5 – Gender Equality	Achieve gender equality and empower all women and girls.
SDG 6 – Clean Water and Sanitation	Ensure availability and sustainable management of water and sanitation for all.
SDG 7 – Affordable and Clean Energy	Ensure access to affordable, reliable, sustainable, and modern energy for all.
SDG 8 – Decent Work and Economic Growth	Promote sustained, inclusive and sustainable economic growth, full and productive employment, and decent work for all.
SDG 9 – Industry, Innovation, and Infrastructure	Build resilient infrastructure, promote inclusive and sustainable industrialization, and foster innovation.
SDG 10 – Reduced Inequalities	Reduce inequality within and among countries.
SDG 11 – Sustainable Cities and Communities	Make cities and human settlement inclusive, safe, resilient, and sustainable.
SDG 12 – Responsible Consumption and Production	Ensure sustainable consumption and production patterns.
SDG 13 – Climate Action	Take urgent action to combat climate change and its impacts.
SDG 14 – Life Below Water	Conserve and sustainably use the oceans, seas, and marine resources for sustainable development.
SDG 15 – Life on Land	Protect, restore and promote sustainable use of terrestrial ecosystems, sustainably manage forests, combat desertification, and halt and reverse land degradation, and halt biodiversity loss.
SDG 16 – Peace, Justice and Strong Institutions	Promote peaceful and inclusive societies for sustainable development, provide access to justice for all and build effective, accountable, and inclusive institutions at all levels.
SDG 17 – Partnerships for the Goals	Strengthen the means of implementation and revitalize the Global Partnership for Sustainable Development.

*Abbreviations*: SDG = Sustainable Development Goal.

### Description of the results

Overall, we identified 617 references through initial searches of bibliographic databases, from which two papers were included (Table 3). Grey literature searches across six websites and search engines generated 31 results, from which ten were included in the review (Table 4).

**Table 3:** Results from peer-reviewed literature searches, showing the efforts under the different Sustainable Development Goals

Authors (Year)	Study Design (Level of Initiative)	SDG	Findings
Devarajan, Prabhakaran, and Goenka (2019) [29]	Non-systematic review of the peer-reviewed and grey literature, with recommendations focused on developing countries. (macro-level)	SDG 3 SDG 10 SDG 11	Recommendations (ambitions) for countries to ensure mobility and dignity of older adults by: • Introducing heat defences that allow walkability for all ages. • Increasing availability of green spaces (connected to target 11.7). • Introducing pedestrian paths that consider age and disability in their design.
Mihnovits and Nisos, (2016) [30]	Non-systematic review of age-based policy frameworks (for housing indicators used to measure housing criteria for older people) and recommendations for housing-specific indicators. (uncategorised)	SDG 11	To capture age-friendly housing, indicators outlined below can be used: • Population by type of living quarters. • Proportion of people who did not have enough money to provide adequate shelter or housing. • Population with access to improved water sources, sanitation facilities, and electricity. • Household in housing units by the type of tenure. • Proportion of people who feel safe in their local area. (The indicators capture qualities under target 11.1).

*Abbreviations*: SDG = Sustainable Development Goal (related to the effort)

Most of the literature consisted of reports from international or national organisations [20–24], although three policy documents [25–27], one policy appraisal [28], and two non-systematic reviews [29, 30] were also included. The literature largely focused on macro- (n = 4) [23–[24, 28]–29] or meso-level (n = 4) [20–22, 31] initiatives; some of the documents provided “recommendations” [25–27] which were difficult to categorise, as they could be applied at multiple levels. All but one documents reviewed existing actions which addressed the SDGs (one document [31] detailed ambitions which, if achieved, would address the SDGs). The majority of documents (n = 7) focused on high-income countries or regions, of which four focused on the United Kingdom [20–22, 31]. One review [29] and two documents [23, 28] discussed the SDGs related to older adults in low- and middle-income countries. Overall, initiatives targeting older adults were mentioned under 12 different SDGs: SDG 1 (*No Poverty*), SDG 2 (*Zero Hunger*), SDG 3 (*Good Health and Wellbeing*), SDG 4 (*Quality Education*), SDG 5 (*Gender Equality*), SDG 8 (*Decent Work and Economic Growth*), SDG 9 (*Industry, Innovation and Infrastructure*), SDG 10 (*Reduced Inequalities*), SDG 11 (*Sustainable Cities and Communities*), SDG 13 (*Climate Action*), SDG 16 (*Peace, Justice and Strong Institutions*), and SDG 17 (*Partnerships for the Goals*). Despite the initiatives being connected to a range of SDGs, there was variation in how commonly particular targets were reported – some papers provided targets for all referenced SDGs, others provided no targets, and a few provided targets for specific SDGs only.

**Table 4:** Results from grey literature searches, showing the efforts under the different Sustainable Development Goals

Document Details	SDG (Targets)	Results	
Authors (year): Fox and Macleod; Cabot Institute for the Environment (2019) [22] Title: Bristol and the SDGs: a Voluntary Local Review of Progress 2019 Initiative type: actions Initiative level: meso	SDG 1 (1.2 and 1.3)	• *Lawrence Weston Community Transport Scheme* provides low-cost transport to older adults and disabled residents to help them travel, socialise, and learn.	
	SDG 10 (10.1 to 10.4)	• The *Bristol Dementia Action Alliance* aims to improve awareness of issues around dementia to make Bristol a dementia-friendly city.	
Authors (year): Bristol City Office (2017) [31] Title: One City Plan and the Sustainable Development Goals Initiative type: ambitions Initiative level: meso	SDG 8 (8.5)	• Establish an *Older people into work* programme to support people aged 65 and over with work, social action, and volunteering to tackle social isolation and age-related poverty by 2019. • Have all older people and disabled people provided with clear pathways back into employment or training by 2037. • Increase the proportion of older people in employment, education, or volunteering by 30.0% from 2018 to 2039.	
	SDG 9 (not stated)	• Ensure that no older people in Bristol are digitally excluded; use digital technologies, activities, and information to support healthy, happy lives.	
	SDG 11 (not stated)	• Ensure that older people in Bristol have access to all available forms of public transport and are confident in getting around the city by 2036. • Ensure that people in Bristol have affordable and easy access to appropriate health, social care, and well-being services within their communities by 2045. • Increase the proportion of older people in employment education or volunteering by 50.0% from 2018 to 2047.	
	SDG 16 (16.1)	• Reduce fear of crime amongst older people in Bristol, so it is in line with the city average so that older people no longer feel disproportionately victimised by 2024.	
Authors (year): Canterbury SDG Forum (2019) [20] Title: Canterbury SDG Forum Reports on local implementation of the Goals Initiative type: actions Initiative level: meso	SDG 3 (3.8)	• *Health and Wellbeing* strategy aimed to ensure early assessment and treatment of people with dementia. • *Ageing Well* strategy launched, which focuses on the provision of facilities for socially stimulating opportunities and activates for older people and offering services to support those with illness and care requirements.	
Authors (year): HM Government (2019) [21] Title: Voluntary national review of the progress towards the Sustainable Development Goals Initiative type: actions Initiative level: meso	SDG 1 (1.1 to 1.3)	• Scotland – *Fairer Scotland for Older People: framework for action* focuses on older people in their communities, service accessibility and financial security. • Wales – *Old People’s Commissioner for Wales and Older People* promotes equality and justice; people aged over 60 have free transport. • England – commitment to guarantee state pension increases.	
	SDG 3 (3.8)	• England – influenza vaccine is available for free for those aged 65 and above.	
	SDG 10 (not stated)	• Northern Ireland – *Healthy Ageing* programme emphasises the needs of older people in policy-making and promotes the use of the Age-friendly Environments in Europe tool for establishing age-friendly programmes.	
	SDG 11 (11.1 and 11.2)	• Scotland – *Affordable Housing Supply Programme* to provide specialist housing so older adults can stay in their homes. • Northern Ireland – *Inclusive Mobility Transport Advisory Committee* to help design accessible and inclusive transport for older people.	
Authors (year): Ministry of Foreign Affairs (2018) [24] Title: Towards a Sustainable and Resilient Singapore Initiative type: actions Initiative level: macro	SDG 1 (not stated)	• The *Central Provident Fund* provides financial security in retirement. • *Silver Support Scheme*, which provides low-income nationals aged 65 and above quarterly cash pay-outs. • *ComCare Long-Term Assistance Scheme* provides financial, medical, and rental assistance to those unable to work due to old age. • *Medifund Silver* provides assistance with medical bills for low-income older adults.	
	SDG 3 (not stated)	• *Agency for Integrated Care* helps pensioners access eldercare services.	
	SDG 10 (not stated)	• *Workfare Income Supplement* scheme supplements the wages of older low-wage workers and tops up their retirement fund.	
	SDG 11 (not stated)	• Transport initiatives (barrier-free entrances, tactile guidance system, and priority queue zones) to guarantee accessibility for older adults. • *Silver Zones* with road safety features to make streets safer for older adults.	
Authors (year): Department of Economic and Social Affairs United Nations (2018) [23] Title: Ageing Related Policies and Priorities in the Implementation of the 2030 Agenda for Sustainable Development – as reported in the Voluntary National Reviews of 2016, 2017 and 2018 Initiative type: actions Initiative level: macro	SDG 1 (1.1 to 1.3)	• More than 40 countries had social services and protection programs (or their expansion) for older persons to reduce their risk of poverty.	
	SDG 2 (2.2 and 2.4)	• Nine country-specific initiatives that address food deprivation and nutrition in older people.	
	SDG 3 (3.3, 3.4, 3.8 and 3.C)	• Older persons considered in prevention and health care strengthening programmes (in more than 28 countries). • 18 countries had plans and programmes targeted at improving the health, care, and wellbeing of older adults.	
	SDG 4 (4.3, 4.4, and 4.6)	• Lifelong learning programmes for older persons rarely reported (only in three countries).	
	SDG 5 (5.2, 5.4, and 5.5)	• Only three countries focused on ageing-related strategies connected to gender equality.	
	SDG 8 (8.5 and 8.8)	• Some schemes (in 16 or more countries) have been launched to encourage the participation of older persons in the labour market.	
	SDG 10 (10.2 and 10.3)	• Social security, research and protection programmes, policies, and social activities for older adults launched in more than 15 countries.	
	SDG 11 (11.2 and 11.7)	• Initiatives that aim to provide safe and affordable transport launched in some states. • City planning to meet the needs of older people, ensuring convenient and efficient access to facilities.	
	SDG 13 (not stated)	• One country has initiated training in natural risk management and relief targeted at older persons.	
Authors (year): United Nations Economic and Social Commission for Western Asia (ESCWA) (2017) [28] Title: Ageing in ESCWA Member States. Third Review and Appraisal of the Madrid International Plan of Action on Ageing (MIPAA) Initiative type: actions Initiative level: macro	SDG 1 (not stated)	• 56.0% of member states report schemes to address poverty, but many are not specific to the needs of older adults.	
	SDG 3 (not stated)	• Many health-related policies and programmes for older persons are targeted at prevention and treatment of non-communicable diseases; home care and mental health programmes are scarce.	
	SDG 4 (not stated)	• 78.0% of the member states have literary policies and programmes that include older persons, but only some target older adults specifically.	
	SDG 8 (not stated)	• 78.0% of the member states encourage early retirement, and 44.0% have schemes to encourage labour-force participation of older adults.	
	SDG 11 (not stated)	• All member states have programmes and policies for age-friendly transport, mobility, or accessibility.	
	SDG 16 (16.6 and 16.7)	• Institutional arrangements on ageing established in all member states, with national committees and strategies adopted in most countries. • All member states have introduced programmes or policies for neglect, violence, and abuse but few specifically protect older adults.	
Authors (year): United Nations Economic Commission for Europe (2020) [26] Title: Gender equality in ageing societies Initiative type: recommendations Initiative level: uncategorised	SDG 5 (not stated)	Recommendations for a life-course approach to ageing and prevention of poverty in women: 1. Prevent gendered disadvantages over the life course through equal entitlements for care-related leave and equal pay for equal work. 2. Reforms to the care sector, like affordable child and eldercare. 3. Pension reforms where periods of informal care are recognised. 4. Reduced gender segregation in occupations and equal pay for equal work. 5. Affordable access to health and social care services and financial support to those with lower income.	
Authors (year): United Nations Economic Commission for Europe (2020) [25] Title: Ageing in sustainable and smart cities Initiative type: recommendations Initiative level: uncategorised.	SDG 1 (not stated) SDG 3 (3.6) SDG 11 (11.4 and 11.7)	1. Allowing ageing in place through collaboration at the city level and flexible homes (which adapt to changes in functionality). 2. Affordable housing through state-funded security systems and subsidised housing sectors. 3. Securing home environments through actions at the home. 4. Increasing access to and availability of green spaces and safe public places, with the involvement of older residents in planning. 5. Ensure that transport is accessible, affordable, safe, and secure through practices like *Sustainable Urban Mobility Plans*, integration of pedestrian requirements, and adaptation of transportation to the needs of older people.	
Authors (year): Stakeholder Group on Ageing (2019) [27] Title: Empowering People and Ensuring Inclusiveness and Equality Initiative type: recommendations Initiative level: uncategorised.	SDG 4 (4.3, 4.6, and 4.7)	• Need for education for older persons, ensuring basic literacy and numeracy, through community initiatives.	
	SDG 8 (8.3)	• Policies that promote gender equality in the labour market and focus on both informal and formal markets are needed. • Anti-age discrimination legislation to reduce inequality in retention, training, and recruitment of older workers. • Removal of age restrictive policies to avoid financial exclusion of older people (to microcredit, loans, and financial investments).	
	SDG 10 (10.1 and 10.3)	• Adequate pensions systems, especially for older women. • Social protection for access to affordable health services. • Policies and programmes to improve political and social participation of older people in communities. • Legal instruments to combat ageism and discrimination and protect the rights of older adults.	
	SDG 17 (17.18)	• Disaggregation of national and regional data by age and other dimensions (sex, disability, and geographic location).	

*Abbreviations*: SDG = Sustainable Development Goal (related to the effort).

### Commonly discussed Sustainable Development Goals

SDG 11, SDG 3, SDG 1, and SDG 10 were the most commonly discussed Goals in the literature. Specifically, nine documents mentioned SDG 11, seven mentioned SDG 3, six mentioned SDG 1, and five mentioned SDG 10.

Recommendations or initiatives targeting SDG 11 frequently considered green spaces, affordability of housing, and accessibility of transport. Walkability was mentioned in a paper specific to low-income countries [29]. Initiatives or recommendations made under SDG 10 were variable and included pension schemes and advocacy as well as programmes for dementia awareness, social protection, and social participation. Efforts under SDG 1 discussed financial assistance, affordable transport, financial security programs (including poverty prevention and pensions), and affordable housing. Conversely, strategies under SDG 3 mostly addressed early treatment and assessment for dementia, preventative medicine, accessibility of care services, and flexible homes for ageing in place.

### Rarely discussed Sustainable Development Goals

Eight other SDGs were mentioned in the literature (Tables 2 and 3); generally, these were not discussed as often (i.e., in more than three papers at a time). In particular, the literature highlighted shortcomings in SDGs 2, 4, 5, and 17 as only some countries provided older adult-specific initiatives that aim to address these goals. A policy paper focused on SDG 5 recommended social care and pension reforms to meet the Goal; the policy brief also considered the importance of affordable services [26]. Goals 13 and 17 were only briefly mentioned; strategies under SDG 17 discussed the need for disaggregated data [27], and those under SDG 13 considered disaster risk management targeted at older adults [23]. Interestingly, one report highlighted the importance of digital inclusion for older people under SDG 9 [31].

### The intersection of the Sustainable Developmen﻿t Goals.

Often, the initiatives and recommendations under individual SDGs overlapped. For example, SDGs 1, 5, and 10 addressed financial security; SDGs 1, 10, and 16 discussed advocating for older people through policy or legislation; and SDGs 5, 8, and 10 mentioned the importance of gender equality. The most commonly discussed efforts concerned the protection of older adults and social participation, with the former appearing across SDGs 1, 8, 10, and 16 and the latter appearing across SDGs 3, 8, 10, and 11. However, not all SDGs intersected in their recommendations, as initiatives under SDGs 2, 4, 9, 13, and 17 did not discuss efforts similar to any other Goals.

### High-income versus low- and middle-income countries

Eight out of twelve sources focused entirely on high-income regions (according to the World Bank classification of economies [32]). Specifically, a document that analysed 111 voluntary national reports from countries of all economic strata did not elucidate differences between low- or middle-income countries and high-income ones in the types of efforts used to address SDGs 1, 2, 3, 8, 10, and 11 (all types of countries had examples of efforts) [23]). For SDGs 4, 5 and 13, no examples from low- and middle-income countries were available; similarly, few examples (three for SDG 4, two for SDG 5, and one for SDG 13) were available from high-income countries [23]. Another document that appraised reports from Western Asian countries considered eight low- and middle-income countries and two high-income ones [28]. With the exception of one targeted anti-poverty programme (in Kuwait), high-income countries did not differ considerably in their strategies, though they did evidence greater participation of older people in policy and programme development (largely through civil society organisations) [28]. A peer-reviewed paper that focused solely on recommendations for “developing countries” discussed how these nations have poor pedestrian facilities and public transport compared to high-income ones [29]. Despite high-income countries having many established free transport schemes and financial security programmes, a focus on SDGs 1, 3, 10, and 11 was still present across many documents [20–22, 24]. Overall, countries within different economic strata did not focus on markedly different goals.

## Discussion

The scoping review has highlighted that schemes, recommendations, or policies concerning older adults were most frequently mentioned under SDGs 1, 3, 10, and 11. Conversely, the literature had limited examples of actions under SDGs 2, 4, 5, and 17. The review summarised the existing efforts that aimed to directly address the SDGs in older populations (in a community setting) prior to the *Decade of Healthy Ageing* and demonstrated the extent of available research.

The documents discussed many types of actions that are needed to improve the lives of older adults as part of the SDGs. For example, financial security, affordable healthcare, accessibility of services, affordable housing, social participation, and inclusion and protection were addressed. These topics are similar to the ones outlined by an important policy document related to ageing and communities, the WHO *Global age-friendly cities: a guide*, which also discusses healthcare, housing, transport, social inclusion, and participation as vital to healthy ageing [33]. These topics are mentioned as part of the eight domains of age-friendly environments, which reflect the physical structures, environment, services, and policies of communities that foster healthy and active ageing [7, 33]. Some other relevant topics were mentioned: with outdoor environments being addressed as part of SDGs 11 and 13 [29], civil engagement and employment as part of SDG 8 [23, 27, 28], and community services and ageing-in-place initiatives as part of the SDG 1 and SDG 11 [25].

However, the *Communication and Information* domain of the WHO framework was not specifically addressed by the literature identified. While the WHO guidelines highlight the importance of clear, accessible information and the need to humanise some automated processes [33], the initiatives under SDG 9 addressed digital inclusion only briefly, and information provision not at all [31].

The actions launched to support the SDGs were generally similar to those discussed under the eight domains of age-friendly environments, exemplifying the continuity within policy on what is required for healthy ageing.

Interestingly, the SDG-targeted actions overlapped significantly. For example, a focus on inclusion and protection of older adults and social participation was present across four (different) SDGs. Likewise, three Goals focused on pensions, financial security, and gender equality. Existing papers have remarked on the interlinkages between the SDGs, noting that some interactions result in co-benefits, and others lead to trade-offs [34, 35]. For example, studies have argued that progress on any Goal is likely to support health, which the UN reiterates through its nexus mapping (pattern of SDG interactions) where SDG 3 is linked to SDG 1, 10, and 11 [34, 36]. Thus, it is unsurprising that the initiatives and recommendations discussed under the various Goals overlap, as the SDGs were designed to relate to and depend on one another [35]. Despite this alignment, efforts may only focus on a single SDG due to fragmented governance, research, and institutions [35], a situation likely influenced by local priorities and funding.

One important finding of the review was that few studies presented SDG-centred schemes specifically targeted at older adults. Some studies excluded from the review did discuss initiatives that target “vulnerable populations” [37–40], a term that aggregates older people, children, women, and people with disabilities. But these four groups potentially have very different needs. Similarly, some of the included reports suggested that general strategies be used to address the SDGs in older populations, but few explicitly targeted older adults. Moreover, some papers discussed age-related strategies in the context of “leaving no one behind” without linking them to a specific SDG [23].

This lack of focus on older adults within SDG-related initiatives is somewhat unsurprising given the limited attention within the SDGs on older adults. Only SDG 2, 3, 10, and 11 make a nod to age or older people within their specific targets. Despite the Goals being designed in a way to “cover issues that affect us all” there is limited appreciation of the unique needs of “vulnerable” groups within them – people with disabilities, children, women, and older adults are often aggregated under the same targets [41]. This lack of focus could result in limited funding for SDG-related programmes targeting older adults, as countries and communities may not prioritise older adults and therefore not invest in programmes related to this group. Moreover, communities may not be able to bid for funding from the UN SDG Fund for projects related to older people, given the lack of focus within the SDG agenda – projects under this fund focus on children or women instead [42]. There are projects under the *Age-friendly Cities and Communities* initiative that can be thought to address the SDGs (without specifically referencing them), given the alignment between the two policy agendas (as demonstrated by the *Decade of Healthy Ageing*). However, these were out of the scope of the review, which aimed to elucidate any programmes *specific* to the SDGs.

Overall, sustainable development projects lack precision and focus on older adults, and the literature centres on high-income regions that are more likely to provide regular national or subnational reports. Two documents that appraised reports by high-, middle- and low-income countries did not elucidate any obvious differences in the types of SDG-based efforts discussed. Nor can in-depth conclusions be reached about discrepancies or similarities between countries of different economic strata, since few reports were available from individual low- or middle-income countries.

### Strengths and limitations

This scoping review has important methodological strengths. The comprehensive search strategy allowed the review to clarify the type of documents available, the extent of the literature, and its nature. Extensive peer-reviewed searches reduced the likelihood of missing any crucial data; while the grey literature searches reduced the risk of publication bias and increased the comprehensiveness of the review [43].

This review does have some methodological shortcomings, such as grey literature searches that focus on reports written in English, shifting the balance towards high-income countries. Ideally, the websites of individual UN bodies, country offices, other age-related and sustainability focused organisations, and reference lists of the policy documents would have been searched to obtain larger samples. This is the object of further research to better understand the alignment, or the lack thereof, between global healthy ageing and sustainability policies.

The scoping nature of the review also limited its analytical depth – although this was not entirely relevant to the aims. The review aimed to showcase the range of literature rather than providing a synthesis of the results. The scope of the review was narrowed to make it relevant in the context of the *Decade* and other healthy ageing policies, which focus on the “community” as a setting. Despite this potentially restricting the volume of literature obtained, “community” was defined in a broad sense, and initiatives at several (meso- and macro-) levels were included.

## Conclusion

The scoping review has revealed useful findings, namely the concentrated efforts targeting older people under SDGs 1, 3, 10, and 11 and the complementary and overlapping nature of the SDG-based efforts. Despite the limited research available on SDG-targeted efforts focussed on older adults, the review findings do align with the eight domains of age-friendly environments. Overall, the review has achieved its aim of summarising the extent, range, and nature of available research. It provides an initial evidence backdrop for future research and policy development that can strengthen the links between healthy ageing and sustainability agendas, and articulates how progress should be monitored.

## Electronic supplementary material

Below is the link to the electronic supplementary material.

Supplementary Material 1

## Data Availability

All data analysed during this study are included in this article. Specifically, data used in this study can be found in the following databases: Scopus, Medline, and Global Health. Also, Google, and the websites of the UN, WHO, Centre for Ageing Better, International Federation of Ageing, and Organisation for Economic Co-operation and Development Search were searched. Search strings are shown in Table 1.
