# Supplementary material for: Efforts to address the Sustainable Development Goals in older populations: a scoping review
Source: BMC Public Health. 2023 Mar 8;23:456. doi: 10.1186/s12889-023-15308-4 (PMC9996959; doi:10.1186/s12889-023-15308-4)
Supplement: Supplementary file 1 — Supplementary Material 1 [file 12889_2023_15308_MOESM1_ESM.docx]

| **Supplementary Table 1**. Scoping review inclusion and exclusion criteria. | |
| --- | --- |
| Inclusion Criteria | Exclusion Criteria |
| - Sample: older adults, as defined by individual studies or documents as the definition varies. - Phenomenon of interest: recommendations, policies, indicators, strategies, or interventions related to the Sustainable Development Goals in the community. - Design: no restrictions were made on the study design. - Evaluation: any outcomes related to the Sustainable Development Goals in older adults (on a community and individual level). - Research type: any study relating to a public health intervention (including, but not limited to, policies, education, and environmental changes). | - Sample: no focus on or mention of older adults. - Phenomenon of interest: no mentions of efforts (recommendations, policies, indicators, strategies, or interventions) to address the Sustainable Development Goals in the community. - Design: no restrictions were made on the study design. - Evaluation: associations, prevalence, or incidence^1^. - Research type: observational studies^1^. |
| ^1^As associations, prevalence, or incidence are not being investigated. | |
